# Neuroimaging for differential diagnosis of transient neurological attacks

**DOI:** 10.1002/brb3.2780

**Published:** 2022-11-09

**Authors:** Ying Wang, Hao Zha

**Affiliations:** ^1^ Department of Neurology The Second Affiliated Hospital of Kunming Medical University Kunming China; ^2^ Department of Reproductive and Genetics The Second Affiliated Hospital of Kunming Medical University Kunming China

**Keywords:** acute ischemic stroke, computed tomography, magnetic resonance image, transient and neurological attack, transient ischemic attack

## Abstract

**Background:**

Rapid yet comprehensive neuroimaging protocols are required for patients with suspected acute stroke. However, stroke mimics can account for approximately one in five clinically diagnosed acute ischemic strokes and the rate of thrombolyzed mimics can be as high as 17%. Therefore, to accurately determine the diagnosis and differentiate mimics from true transient ischemic attacks, acute ischemic stroke is a challenge to every clinician.

**Discussion:**

Medical history and neurological examination, noncontract head computed tomography, and routine magnetic resonance imaging play important roles in the assessment and management of patients with transient neurological attacks in the emergency department. This review attempts to summarize how neuroimaging can be utilized to help differentiate the most common mimics from transient ischemic attack and acute ischemic stroke.

**Conclusion:**

Although imaging can help direct critical triage decisions for intravenous thrombolysis or endovascular therapy, more detailed medical history and neurological examination are crucial for making a prompt and accurate diagnosis for transient neurological attack patients.

## Graphical abstract

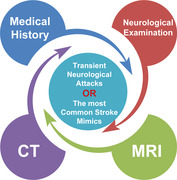

## 1 INTRODUCTION

Transient neurological attacks (TNAs) are common causes of emergency department (ED) visits. The final diagnosis of patients with TNAs can be divided into three categories: transient ischemic attack (TIA), acute ischemic stroke (AIS), and mimics (Mimics) after obtaining medical history, neurological examination, laboratory tests, and neuroimaging. As the risk of stroke recurrence or progression is highest in the 90 days following the first episode of either TIA or AIS, these two causes are of greatest concern. However, focal ischemia is not the cause of most TNAs. According to the literature, Mimics account for 18–60% of all patients with TNA (Amort et al.; 2011, Dutta et al.; 2015, Yu et al.; 2017, Kwok et al.; 2014, Sadighi et al.; 2019, Prabhakaran et al.; 2008, Ferro et al.; 1996, Nadarajan et al.; 2014, Sadighi et al.; 2019, Gioia et al.; 2016, Mackay et al.; 2016) (Table 1).

**TABLE 1:** Percentage of TIA, AIS, and mimics in different studies

Literatures	TIA	AIS	Mimics
Amort et al. (Amort et al.; 2011)	248 (81.8%)		55 (18.2%)
Dutta et al. (Dutta et al.; 2015)	337 (31.6%)	189 (18%)	538 (50.4%)
Yu et al. (Yu et al.; 2017)	163 (39.1%)	155 (37.2%)	99 (23.7%)
Kwok et al. (Kwok et al.; 2014)	1131 (35.1%)	665 (20.6%)	1426 (44.3%)
Sadighi et al. (Sadighi et al.; 2019)	57 (22.4%)/Possible TIA 90 (35.4%)		107 (42.1%)
Prabhakaran et al. (Prabhakaran et al.; 2008)	40 (40%)		60 (60%)
Ferro et al. (Ferro et al.; 1996)	4 (13%)	10 (32%)	17 (55%)
Nadarajan et al. (Nadarajan et al.; 2014)	Definite or possible TIA 1148 (75%)	46 (3%)	338 (22%)
Sadighi et al. (Sadighi et al.; 2019)	131 (50.6%)	31 (12.0%)	97 (37.4%)
Gioia et al. (Gioia et al.; 2016)	66 (12.2%)	208 (38.2%)	416 (43.2%)
Mackay et al. (Mackay et al.; 2016)	10 (3.6%)	55 (19.7%)	84 (30.1%)

Accurate diagnosis is the premise for developing an individualized treatment for patients. Misdiagnosis causes the patient to receive the wrong treatment, for example, patients with Mimics may be treated with intravenous tissue plasminogen activator (tPA) which adds additional risks of complications. Although the literature only contains a few cases of patients with symptomatic intracranial hemorrhage receiving thrombolysis (Nguyen & Chang, 2015) or mentions that tPA administration in mimics is safe (Giraldo et al.; 2011, Martinez‐Ramirez et al.; 2010), it can still cause potential harm to these patients. For instance, the economic burden (Goyal et al.; 2015), including excess hospital costs, indirect financial burden, and intangible costs, has incurred various negative effects on the family of patients. In addition, side effects caused by tPA may bring a series of risks, including bleeding (Giraldo et al.; 2011, Mansoor et al.; 2013)—specifically, minor bleeding complications like gingival bleeding remains a serious threat. Therefore, the identification and differentiation of TIA, AIS, and Mimics help clinicians to perform reasonable postadministration of patients, as well as effectively assign public resources to improve patient prognosis, with significant effect in clinical practice.

The major manifestations of TNA with Mimics are classified as unilateral lime weakness, asymmetric visual field defect, dysarthria, facial droop, unilateral limb numbness, facial numbness, sudden dizziness, diplopia, speech disturbance, headache, loss of consciousness, and seizure‐like activity. Magnetic resonance imaging (MRI) is more sensitive than computed tomography (CT) in identifying infarct lesions in patients with a clinical history of TIA. In patients with a clinical history of TIA, abnormal hyperintensity can be found on diffusion‐weighted imaging (DWI) 1–2 h after the onset of symptoms. The frequency of the abnormally high signal increases with the duration of symptoms (Amort et al.; 2011, Lee & Frayne, 2015). In addition, CT angiography (CTA) is now the standard tool in CT‐based stroke workup for the detection of large vessel occlusion (LVO) and overall assessment of the vasculature including the neck. CTA has a distinct advantage that it is fast, easy to obtain, provides a comprehensive view of the arterial system, and has high accuracy in identifying LVO (Lee & Frayne, 2015). From 10 previous studies (*n* = 1136) (Amort et al.; 2011, Prabhakaran et al.; 2008, Nadarajan et al.; 2014, Sadighi et al.; 2019, Lee & Frayne, 2015, Noureddine et al.; 2014, Ghia et al.; 2010, Kozera‐Strzelinska et al.; 2019, Paolini et al.; 2013, Jiang et al.; 2014), the 16 most common and alternative final diagnoses of patients with Mimics are given in Table 2.

**TABLE 2:** Main alternative discharge diagnoses for mimics

Final diagnosis of mimics	Number	Percentage
Migraine	305	26.8%
Vertigo and dizziness	110	9.7%
Syncope	105	9.2%
Epileptic seizures	103	9.1%
Psychiatric	91	8.0%
Toxic/metabolic	85	7.5%
Neuropathy	76	6.7%
Neoplasm of the brain	44	3.9%
Infection	43	3.8%
Transient Global amnesia	35	3.1%
Dementia	33	2.9%
Cardiac condition	18	1.6%
Alcohol and drugs	17	1.5%
Hemorrhage	6	0.5%
Hypoglycemia	3	0.3%
Others	62	5.5%

In the ED, clinicians need to diagnose patients with TNAs as soon as possible by using a combination of medical history and neurological examinations with neuroimaging to avoid delayed treatment. CT and MRI are increasingly available for rapid assessment of TNAs. In recent years, new methods of CT and MRI (e.g., fMRI) have rapidly developed and been applied in more clinical fields. However, implementation in clinical practice faces the following problems. First, the new methods of CT and MRI are unlikely to be applied to ordinary emergency patients. Second, most hospitals can only perform routine imaging. Moreover, timing is crucial, and thus the time that it takes to obtain effective information or results from imaging can be a limitation. Finally, given the huge number of patients in the ED, it will inevitably increase the economic burden on the government, hospitals, and individuals due to performing indiscriminate imaging examinations. Therefore, it is imperative to establish proper criteria for selecting imaging examinations to effectively control medical expenses and requires urgent problem‐solving.

The most common imaging methods of CT and MRI include noncontrast CT, CTA, CT perfusion (CTP), T1‐weighted imaging (T1WI), T2WI, fluid‐attenuated inversion recovery (FLAIR), DWI, and perfusion‐weighted imaging (PWI). This study aimed to select the most common and effective imaging methods by a literature review to assist clinicians in the diagnosis of patients with TNA (Table 3).

**TABLE 3:** Key points of differential diagnosis of mimics from TNA

Final diagnosis of mimics	Key points of differential diagnosis
Migraine	1. The periaqueductal gray volume expansion and dysfunction can be recognized in MRI scans. 2. WHMs are often found in patients with migraine, especially women. 3. PWI is remarkably accurate for acute migraines. 4. There is a significant difference between the hypoperfusion sites in patients with TIA and AIS, which is usually limited to specific vascular territories responsible for the symptoms, and the increasing TTP or MTT.
Vertigo and dizziness	1. Medical history and physical examination are still the most important tools to diagnose patients with vertigo and dizziness. 2. The Dix–Hallpike and Roll tests are effective diagnostic methods for benign paroxysmal positional vertigo. 3. DWI‐MRI is the best choice for suspected central vertigo caused by basilar and vertebral occlusions.
Syncope	In patients with syncope, SPECT images showed significantly decreased CBF in the right anterior insular cortex, left para‐hippocampal gyrus, bilateral fusiform gyri, bilateral middle and inferior temporal gyri, left lingual gyrus, bilateral precuneus, and bilateral posterior lobes of the cerebellum.
Epileptic seizures	1. Early use of CTP to determine different perfusion patterns, especially local hyperperfusion, can be differentiated from AIS in patients with acute focal neurological deficit symptoms, such as aphasia, hemiplegia, and gaze. For epileptic seizures, the temporal and parietal lobes are most prone to perfusion changes and generally do not conform to vascular distribution. 2. MRIs of patients with epileptic seizures may show hyperintensity as well as mild swelling of the cortical gray matter and subcortical white matter (not necessarily around a vascular territory) on T2WI and FLAIR imaging.
Psychiatric	1. Neuroimaging in psychiatric disorder rarely reveals any remarkable structural brain abnormalities. 2. Conventional history‐taking, mental status, and neurological examination remain the most important tools for diagnosis.
Toxic/metabolic	Detailed medical history and necessary tests to identify toxic substances. Carbon monoxide (CO) poisoning: This presents with diffuse hypodensity of the gray matter on CTs, high signal on DWIs, and low apparent diffusion coefficient (ADC) values (used to distinguish benign from malignant lesions) in the cerebral cortex and basal ganglia. Methanol poisoning: MRI sensitively demonstrates preferential involvement of both putamina, which shows isointensity on T1WI and hyperintensity on T2WI. T2WI is particularly sensitive to the water content of brain tissue and can also detect a bilateral edema signal in the external capsule.
Neuropathy	The lesion side in the acute phase of Bell's palsy can reliably be identified via higher signal intensity in the geniculate ganglion, labyrinthine segment, and tympanic segment using T1WI.
Neoplasm of the brain	1. On MRI, low and uniform signals on T1WI images, a central hypointensity on T2WI images correlating with necrosis, and a perifocal hyperintensity on T2WI and FLAIR images correlating with edema or noncontrast‐enhancing tumors. 2. The density of a brain tumor hemorrhage on a CT scan is lower than that of a hemorrhage.
Infection	MRIs revealed posterior reversible encephalopathy syndrome, WMHs, brain atrophy, and perivascular space edema.
Transient global amnesia	No visible abnormality was seen using T1WI, T2WI, or FLAIR images 48−74 h after demonstrating increased isolated signal in the hippocampus (or both sides) via an MRI‐DWI test and decreased ADC value.
Dementia	1. Atrophy can be assessed by T1WI. 2. Imaging scales (e.g., the Fazekas scale) can be used in quantitative evaluation.
Cardiac condition	Reliable electrocardiography is a simple test to assist in diagnosis.
Alcohol and drugs	1. In cases of Wernicke encephalopathy, the bilateral and symmetrical hypodensities on CT, hyperintensities on T2WI, and FLAIR images in the periventricular thalami and mammillary bodies can be seen. 2. Heroin‐induced leukoencephalopathy, also known as “chasing the dragon,” diffuse brain lesions involved in the corpus callosum, bilateral thalamus, basal ganglia, radial crown, central semioval, symmetric hypointensities on T1WI, and hyperintensities on T2WI in the periventricular white matter are seen.
Hemorrhage	1. The preferred imaging method is CT. 2. In the super‐acute phase, a hemorrhage presents as a low signal on T1WI, or a high signal on T2WI, FLAIR, and DWI within 24 h. 3. In the acute phase, all of the above sequences present as low signals within 1–3 days.
Hypoglycemia	The imaging mainly characterizes cerebral edema with a high signal on T2WI, FLAIR, and DWI, or a low ADC value in the parietal lobe, occipital lobe, hippocampus, globus pallidus, and striatum—a condition which is reversible and disappears with the recovery of blood glucose.

## 2 MIGRAINE

A migraine is the most common condition among patients with Mimics. A migraine has characteristic features associated with acute episodic symptoms, often lasting 4−72 h. Identifying reliable neuroimaging features of migraines can be useful in daily clinical practice for differentiation from TIA or AIS to guide accurate diagnosis and precise treatment. Among the brain structures of patients with migraines, the periaqueductal gray volume expansion and dysfunction can be recognized in MRI scans, especially in episodic migraines (Chen et al.; 2017). Although there is no explicit evidence of an association between white matter hyperintensities (WMHs) and migraines, WHMs are often found in patients with migraine, especially women. WMHs are mostly distributed in the subcortical white matter of the frontal lobe (Seneviratne et al.; 2013). Even if no obvious abnormal signs can be seen on DWI images of patients with migraines (Adam et al.; 2018), a clinician should consider migraine as a possible diagnosis if the patient has a history of migraines (Lima et al.; 2019). PWI is remarkably accurate for acute migraines, especially among patients with migraine aura. In 70% of migraine aura cases, a significant hypoperfusion occurs in more than one arterial territory, and is not limited to the territory of a specific artery but affects two or more vascular territories even in the bilateral cerebral hemisphere (Adam et al.; 2018, Forster et al.; 2014, Floery et al.; 2012). Furthermore, there is a mismatched relationship between the symptoms and hypoperfusion sites. Hypoperfusion has indicative patterns, including delayed mean transit time (MTT) and time–to–peak (TTP), decreased ratio of TTP/MTT, decreased cerebral blood flow (CBF), and minimal decreased cerebral blood volume (Forster et al.; 2014, Charidimou et al.; 2017). There is a significant difference between the hypoperfusion sites in patients with TIA and AIS, which is usually limited to specific vascular territories responsible for the symptoms, and the increasing TTP or MTT. MRIs can be useful when used together with the detailed medical record of patients, and careful neurological examination for early detection of migraine aura from TIA and AIS (Adam et al.; 2018).

## 3 VERTIGO AND DIZZINESS

Vertigo and dizziness are common symptoms among patients with ED visits and are caused by two categories of diseases: peripheral and central vestibular disease (Han et al.; 2017). CTs and MRIs are frequently used to assist in the diagnosis and cause of vertigo and dizziness; however, clinical studies have demonstrated that neuroimaging is too costly and a low‐yield approach due to mostly negative findings. For instance, only 10% of people presenting with vestibular symptoms have abnormalities on CTs and MRIs (Quimby et al.; 2018, Fakhran et al.; 2013, Ammar et al.; 2017, Honda et al.; 2014, Lawhn‐Heath et al.; 2013), particularly for negative cases of the vestibular peripheral disease. Furthermore, in Meniere's disease, MRIs of the inner ear can detect endolymphatic hydrops to assist in the diagnosis. However, the criteria for endolymphatic hydrops have not been unified nor is MRI of the inner ear a routine imaging method. Therefore, using neuroimaging for patients with peripheral vertigo and dizziness mostly serves to narrow down the differential diagnosis (Craighero et al.; 2011). In this regard, medical history and physical examination are still the most important tools to diagnose patients with vertigo and dizziness (Han et al.; 2017, Ammar et al.; 2017). We emphasize improved application and interpretation of the neurological examination which is of great value in localizing the central and peripheral vestibular lesions. Benign paroxysmal positional vertigo (BPPV) is the most common peripheral vestibular disease (Kroenke et al.; 2000). The Dix–Hallpike and Roll tests are effective diagnostic methods for BPPV that are economical and simple to perform at the bedside (Kim & Zee, 2014). Moreover, the HINTS test (Head Impulse, Nystagmus, Test of Skew) has a higher sensitivity and specificity than early neuroimaging for reliably predicting central vertigo caused by posterior circulation stroke (Quimby et al.; 2018, Ammar et al.; 2017, Kattah et al.; 2009, Newman‐Toker et al.; 2013). However, it is necessary to perform neuroimaging when a patient presents with either vertigo or dizziness to eliminate the possibility of AIS, including imbalance, limb ataxia, gait abnormality, vertical or rotational nystagmus, lateral skew, and when showing no improvement using standard antivertiginous treatment (dimenhydrinate/betahistine) (Ammar et al.; 2017, Honda et al.; 2014, Akoglu et al.; 2018). In addition, noncontrast CT is feasible in the diagnosis of basilar and vertebral stroke but lacks sensitivity and specificity due to the bony posterior fossa artifact and lower image quality. Therefore, DWI‐MRI is arguably the best choice for suspected central vertigo caused by basilar and vertebral occlusions (Lawhn‐Heath et al.; 2013, Akoglu et al.; 2018, Hixson et al.; 2016, Hanna et al.; 2019). Clinicians need to be properly trained in basic clinical examination, which reduces the high cost associated with imaging and unnecessarily elevates the threshold for imaging patients with acute vertigo and dizziness. Distinguishing vertigo and dizziness from posterior circulation TIA and AIS is one of the most important diagnostic challenges.

## 4 SYNCOPE

Syncope is a symptom that presents with an abrupt, transient, complete loss of consciousness due to transient global cerebral hypoperfusion. It is associated with an inability to maintain a postural tone followed by rapid and spontaneous recovery (Hutt‐Centeno et al.; 2019). The European Society of Cardiology has classified syncope into three main categories: reflex (neuronally mediated) syncope, syncope due to orthostatic hypotension, and cardiac syncope (Moya et al.; 2009). As syncope is a transient symptom that occurs and recovers quickly, it is easily confused with TIA. The American Academy of Neurologists state that a TIA is caused by “focal brain…ischemia” and not “global hypoperfusion” (Mansoor et al.; 2013, Easton et al.; 2009). Therefore, the critical point in identifying syncope and TIA is that TIA is only a focal ischemic event, but syncope is caused by global hypoperfusion, which is confirmed by single‐photon emission computed tomography (SPECT). In patients with syncope, SPECT images showed significantly decreased CBF in the right anterior insular cortex, left para‐hippocampal gyrus, bilateral fusiform gyri, bilateral middle and inferior temporal gyri, left lingual gyrus, bilateral precuneus, and bilateral posterior lobes of the cerebellum (Joo et al.; 2011). However, SPECT is not routinely ordered in the evaluation of syncope. In most cases, global perfusion was evaluated by TCD instead of CTP and PWI on the insistence of neurologists not to use routine neuroimaging to evaluate syncope (Shen et al.; 2017). Noncontrast CT is widely used in the evaluation of patients with syncope for two reasons. First, the cause of syncope is diverse. Clinicians may, therefore, try to obtain some clues from neuroimaging for diagnosis. Second, syncope presents with a higher risk of fall‐related injuries (Numé et al.; 2018), in which case head trauma needs to be determined. However, patients with syncope will not benefit from neuroimaging solely by a literature review (Hutt‐Centeno et al.; 2019, Shen et al.; 2017, İdil & Kılıc, 2019, Goyal et al.; 2006, Sclafani et al.; 2010, Gebreselassie et al.; 2016). More detailed medical history, including the triggering factors and presyncope symptoms, is required to determine a holistic picture of patients for accurate diagnosis.

## 5 EPILEPSY

Epilepsy is one of the most frequent acute ischemic event mimics (Adam et al.; 2018). It is difficult to diagnose patients based on their onset of seizures in the ED, especially in an acute situation without family witnesses nor a medical history of seizures available. The preferred imaging modalities in epilepsy are CTs and MRIs (Fonseca Hernández et al.; 2018, Ben Ameur et al.; 2014, Peng, 2019). During seizures, epileptogenic foci neuronal electrical activity is accompanied by characteristic perfusion changes. Early use of CTP to determine different perfusion patterns, especially local hyperperfusion, can be differentiated from AIS in patients with acute focal neurological deficit symptoms, such as aphasia, hemiplegia, and gaze. For epileptic seizures, the temporal and parietal lobes are most prone to perfusion changes and generally do not conform to vascular distribution. Hyperperfusion mainly occurs in the cortex, and most of the deep white matter is not involved. However, compared with low perfusion, the insula appeared more frequently. In emergency situations, MRI‐based diffusion‐weighted and perfusion imaging can also be used to assist in the identification of AIS (Peng, 2019). Nevertheless, MRIs of patients with epileptic seizures may show hyperintensity as well as mild swelling of the cortical gray matter and subcortical white matter (not necessarily around a vascular territory) on T2WI and FLAIR imaging (Torres et al.; 2018), which are not neuroimaging features of epileptic seizures. Instead, they might reveal major acute pathologies that need urgent treatment (Duncan, 2019) and might expose whether the seizure was generated by a gross or subtle structural brain abnormality (Bonilha et al.; 2004, Jackson et al.; 1996, 2006), such as neoplasms, trauma, hemorrhage, large infarct, or arterial‐venous malformations. In these instances, a necessary exam is scheduled for further investigation combined with brain imaging.

## 6 PSYCHIATRIC DISORDER

A psychiatric disorder in patients with TNAs is more common in women, individuals with lower socioeconomic status, and those with lower education levels. In fact, the most common causes are hyperventilation, conversion disorder, somatoform disorder, depression, and anxiety. Many of these patients cannot cooperate during the relevant physical examination by a physician, making accurate diagnosis difficult, especially in patients presenting with their first episode of a psychiatric disorder. Studies have revealed that physicians often order CTs and MRIs for these patients primarily for fear of missing a potentially treatable cause of psychosis or serious neurological illness; however, neuroimaging in these instances rarely reveals any remarkable structural brain abnormalities (LeBaron et al.; 2019, Forbes et al.; 2019, Falkenberg et al.; 2017, Robert Williams et al.; 2014). Therefore, conventional history‐taking, mental status, and neurological examination remain the most important tools for diagnosis.

## 7 TOXIC/METABOLIC

The onset of acute poisoning/metabolic diseases is mostly caused by chemicals, toxic gases, biological toxins, and food poisoning. For diagnosis, in addition to a detailed medical history and necessary tests to identify toxic substances, abnormal manifestations during imaging can be helpful to clinicians.

Carbon monoxide (CO) poisoning and methanol poisoning are the most common poisoning/metabolic diseases in ED visits. CO poisoning is divided into two stages: an acute stage and delayed encephalopathy. Patients with CO poisoning who are sent to the ED with TNA as the main manifestation usually have a short onset time; thus, most patients are still in the acute stage. This presents with diffuse hypodensity of the gray matter on CTs, high signal on DWIs, and low apparent diffusion coefficient (ADC) values (used to distinguish benign from malignant lesions) in the cerebral cortex and basal ganglia. Interestingly, white matter is relatively spared. It is also important to focus on the change of bilateral globus pallidus in patients suspected of CO poisoning. The globus pallidus is the most common site of involvement in CO poisoning (Saini et al., 2014, Raschilas et al.; 2002) and showed symmetrical low density on both sides of the basal ganglia (Kanaya et al.; 1992). This can be seen more clearly on MRIs than on CTs, and present as T1WI hypointensities as well as T2WI and FLAIR hyperintensities in the medial portions of the globus pallidus. Some scans may have demyelinating changes not characteristic of CO poisoning (Torres et al.; 2018, Sung et al.; 2018, Lo et al.; 2007, Hantson & Duprez, 2006).

Acute methanol poisoning is usually caused by drinking fake alcohol with excessive methanol or mistakenly taking methanol as ethanol. Damage to the central nervous system and eyes are the most common symptoms. The toxic effect of methanol is caused by the methanol itself and its metabolites formaldehyde and formic acid. Basal ganglia involvement is likely because of the direct effect of methanol metabolites and the selective vulnerability of the basal ganglia to acidosis (Jain et al.; 2013). This can be reflected in hyperintensities on DWI as similar radiologic findings as cyanide poisoning (Peters et al.; 2007); hence, it cannot be used as a typical imaging feature of acute methanol poisoning alone. According to the literature, the characteristic lesion of acute methanol poisoning is the putamina of the basal ganglia region (Torres et al.; 2018, Hantson & Duprez, 2006, Jain et al.; 2013, Peters et al.; 2007, Arora et al.; 2007), which presents with symmetrical decreased density and a kidney‐like shape. MRI sensitively demonstrates preferential involvement of both putamina (Hantson & Duprez, 2006), which shows isointensity on T1WI and hyperintensity on T2WI. T2WI is particularly sensitive to the water content of brain tissue and can also detect a bilateral edema signal in the external capsule. Moreover, hemorrhages are also observed in patients with severe methanol poisoning who show the high signal intensity of hemorrhagic foci on T1WI. Another characteristic symptom in the early stage of the acute phase is optic nerve edema which presents as a high signal within a hypointensity in the coronal plane during T2WI (Peng et al.; 2005). When symmetrical lesions are detected in the basal ganglia along with sudden visual disturbance, methanol intoxication should be considered in the diagnosis.

## 8 PERIPHERAL NEUROPATHY

Peripheral neuropathy originates as nerve damage in the extremities of the body, such as the face, hand, and feet. A facial droop along with numbness or weakness in the hands or feet is one of the most common symptoms in patients with peripheral neuropathy. Peripheral neuropathy is mostly gradual during chronic development but can be acute, which is easily confused with AIS, such as Bell's palsy (idiopathic facial paralysis) as a common misdiagnosis. The pathological changes in Bell's palsy are mainly neuroedema and demyelination, and in most cases, the MRI is usually not ordered in clinically obvious cases of Bell's palsy (Al‐Noury & Lotfy, 2011). Although the lesion side in the acute phase of Bell's palsy can reliably be identified via higher signal intensity in the geniculate ganglion, labyrinthine segment, and tympanic segment using T1WI (Al‐Noury & Lotfy, 2011, Song et al.; 2008, Seok et al.; 2008, Burmeister et al.; 2011, Yetiser et al.; 2003), it remains a complicated procedure involving mandatory contrast agent injection under emergency conditions. Typical Bell's palsy presents with a characteristic peripheral facial paralysis sign which can be distinguished from central facial paralysis. In addition, MRI may play a role in excluding other facial nerve lesions for the atypical presentation of Bell's palsy, such as gradual‐onset palsy not partially recovering with time and recurrent palsy (Al‐Noury & Lotfy, 2011, Zhang et al.; 2007). The occurrence of tumors should be distinguished and is usually focused on the lesion of the temporal bone, internal acoustic canal, and/or cerebellopontine angle (Alaani et al.; 2005) in the MRI of atypical patients with Bell's palsy.

## 9 BRAIN NEOPLASMS

Brain neoplasms occasionally present with sudden onset of symptoms similar to those seen with TIA or AIS, which are also known as tumor attacks (Liu et al.; 2013). The differential diagnosis of brain tumor attacks and TIA or AIS is crucial since their treatment is quite different. Glioblastomas, meningiomas, and metastases are the most common intracranial tumors masquerading as acute strokes (Liu et al.; 2013). The neuroimaging of glioblastomas can identify the characteristics of mass effect, compression of the surrounding peritumoral tissue, and midline deviation (Shukla et al.; 2017), which present mostly low and uniform signals on T1WI images, a central hypointensity on T2WI images correlating with necrosis, and a perifocal hyperintensity on T2WI and FLAIR images correlating with edema or noncontrast‐enhancing tumors (Wirsching et al.; 2016). Glioblastomas are usually characterized by hyperintensity on DWI and decreased value on ADC due to the presence of concentrated tumor cells and small extracellular space. DWI of patients with AIS also shows high signal lesions that would gradually decrease. However, in the earlier phases of AIS, no obvious differences can be seen with T1WI, T2WI, and FLAIR images, which is inconsistent with the MRI appearance of glioblastomas. The neuroimaging presentation of metastases is hypointense on T1WI images and hyperintense on T2WI MRI. Multiple lesions with heterogeneous rings occur commonly at the gray and white matter junction (Fadili et al.; 2017), and the brain metastases occur predominantly in the middle cerebral artery region. Moreover, the volume of the metastases can be small, but the peritumoral edema appears patchy on T2WI images, thus referred to as “small tumor large edema” (Cha, 2006, Cha et al.; 2007, Law et al.; 2002, Cha et al.; 2002, Cha, 2004, Law et al.; 2003). Meningiomas are mostly located outside the brain parenchyma, in the sagittal sinus, cerebral convex surface, cerebellopontine angle, cerebral palsy, or cerebellum. Their signals on T1WI and T2WI images are similar to the cortical signals of adjacent brain tissue, while, as opposed to AIS, these signal levels occur in the middle of the intensity range. To identify brain neoplasms, contrast‐T1WI could be considered as necessary. In addition, during the growth process of brain neoplasms, a large number of immature blood vessels are produced within the brain tumor tissue, which can easily experience a rupture and cause intracranial hemorrhage. This event can potentially be misdiagnosed as hypertensive hemorrhage on a noncontrast CT. A hemorrhage that originated from intracranial tumor tissue is often located in the brain lobes, while hypertensive hemorrhages are predominantly found in the basal ganglia (Tsuchiya et al.; 2015). The shape of a hemorrhage is irregular, while the shape of a hypertensive hemorrhage is oval with clear edges and easily rupture into the ventricles. The density of a brain tumor hemorrhage on a CT scan is lower than that of a hemorrhage. The density is unified as a result of the necrosis, cystic changes, and calcification components inside the tumor. Due to the space‐occupying effect of brain tumors, the edemas that form around the hemorrhage as results are more extensive and can last longer (Kumar et al.; 2011).

## 10 TRANSIENT GLOBAL AMNESIA

Transient global amnesia (TGA) is a sudden‐onset of anterograde memory loss. It occurs with no change of consciousness or loss of self‐awareness, as well as no neurological symptoms besides dizziness or headaches. Furthermore, these symptoms are spontaneously resolved after 1–24 h (Hodges & Warlow, 1990). In most cases of TGA, MRI indication may be negative in the first few hours after the initial attack [100]; however, reviewing the retrospective analysis, patients with TGA underwent a prescribed MRI after demonstrating increased isolated signal in the hippocampus (or both sides) via an MRI‐DWI test and decreased ADC value. Simultaneously, no visible abnormality was seen using T1WI, T2WI, or FLAIR images 48−74 h after the initial symptoms. A follow‐up MRI at 2 months was also normal (Pearce et al.; 2018, Jain et al.; 2018, Förster et al.; 2017, Wilkinson et al.; 2013, Matsui et al.; 2002, Toledo et al.; 2008, Hashimoto et al.; 2019, Woolfenden et al., Nov 1997). Based on the above imaging findings, for patients who are suspected to have TGA in the ED, clinicians need to consider the interval time between the symptom onset and the MRI scheduled though most patients usually do not reach 48 h in the ED (Rhodes et al.; 2016, Kocher et al.; 2012). Therefore, considering above the issues, the diagnosis of TGA should be based on clinical features.

## 11 CARDIAC CONDITION

Acute myocardial infarction, including arrhythmias, are common cardiac conditions in the ED (Wang et al.; 2018). Due to insufficient cardiac output leading to cerebral hypoperfusion, the underlining cardiac condition may be the first symptom diagnosed in patients with TNAs often resulting in misdiagnosis. Therefore, clinicians should be aware of the high likelihood of cardiac diseases with TNAs. In addition to comprehensive medical history and detailed physical examination, reliable electrocardiography is a simple test to assist in diagnosis.

## 12 ALCOHOL/DRUG ABUSE

Alcohol is one of the most abused substances, and as a result, alcohol poisoning may be a cause of TNA. In patients with acute alcohol poisoning, routine noncontrast CT scans are recommended in the course of diagnosis, including in coma patients with a history of head trauma without a witness, focal neurological signs detected when the amount and concentration of alcohol are not consistent with the lack of consciousness, and in those who experience worsening condition after routine naloxone treatment for 2 h (2014). Global volume loss, marked diffuse cerebellar atrophy, and alcohol‐induced changes are often observed in routine CT or MRI scans of patients with alcohol addictions (Geibprasert et al.; 2010). In addition, encephalopathy associated with alcoholism also needs to be considered (e.g., Wernicke encephalopathy). In cases of Wernicke encephalopathy, the bilateral and symmetrical hypodensities on CT, hyperintensities on T2WI, and FLAIR images in the periventricular thalami and mammillary bodies can be seen (Gallucci et al.; 1990, Weidauer et al.; 2003).

Other common substances of drug abuse include opium and cocaine (Dines et al.; 2015, Orsini et al.; 2017, Liakoni et al.; 2017). Heroin abuse is the leading cause of primary treatment admissions and acute opioid toxicity presentation (Tropea et al.; 2017). Typical MRI findings for this type of abuse include symmetric hyperintensities on T2WI and FLAIR images in both globi pallidi and the cerebellum (Alambyan et al.; 2018, CAI et al.; 2000). Heroin‐induced leukoencephalopathy, also known as “chasing the dragon,” diffuse brain lesions involved in the corpus callosum, bilateral thalamus, basal ganglia, radial crown, central semioval, symmetric hypointensities on T1WI, and hyperintensities on T2WI in the periventricular white matter are seen (Xu et al.; 2002, Abdilla et al., 27 2019). The acute neurovascular lesion related to cocaine abuse also needs to be differentiated from acute ischemic events. Cocaine overdoses may induce large cerebral infarction regions showing diffusion restriction (Peterson et al.; 1991). Although these imaging findings are nonspecific, when combined with the medical history of a patient, they can effectively help clinicians make a diagnosis.

## 13 INFECTION

Infections occurring in other organs, such as urinary tract infections, pneumonia, otitis media, and sinusitis, may also be misdiagnosed as Mimics (Amort et al.; 2011, Ghia et al.; 2010). Some sepsis cases caused by severe infections can also lead to acute brain dysfunction with consciousness disorder and focal neurological signs. In these instances, MRIs revealed posterior reversible encephalopathy syndrome, WMHs, brain atrophy, and perivascular space edema (Esen et al.; 2020, Sutter et al.; 2015, Fugate et al.; 2010). However, the relationship between these imaging results and acute brain dysfunction caused by sepsis is not yet clear and requires further research.

## 14 DEMENTIA

At least 20% of elderly patients in ED visits have cognitive impairment or dementia (O'Sullivan et al., 2018, Clevenger et al.; 2012). As there is a significant loss in memory, learning, orientation, understanding, judgment, calculation, and language function of these patients, clinicians encounter more difficulties in collecting their medical history and performing a physical examination. Therefore, brain imaging is mandatory for differential diagnosis of patients considered with dementia (Jia et al.; 2018). In emergency conditions, brain structure information can be obtained by T1WI, whereas brain parenchymal abnormalities can be seen by T2WI and FLAIR, which facilitate clinicians to quickly and accurately identify and distinguish dementia from other TNAs. Alzheimer's disease is the most common type of dementia, with a characteristic feature of medial temporal lobe atrophy as diagnostic evidence, and affects primarily the hippocampus and entorhinal cortex (2019, Valkanova & Ebmeier, 2014). Vascular dementia is caused by cerebrovascular disease, which is also easily confused with acute ischemic events in ED visits. Furthermore, small vessel disease is the most common cause of vascular dementia (Pantoni, 2010), with different MRIs capable of revealing WMHs, subcortical infarcts, lacunas, perivascular spaces, cerebral microbleeds, and atrophy. For instance, high WMHs can be seen on T2WI and FLAIR images; subcortical infarcts can be identified on DWI; typical lacunas are characterized by high signals on T2WI, as well as high signals on the periphery and low signals on the core via FLAIR; most cases of perivascular space are linear without a hyperintense rim on T2WI; cerebral microbleeds require susceptibility‐weighted imaging (SWI) imaging though often impossible in emergencies; and atrophy can be assessed by T1WI (Wardlaw et al.; 2013). Moreover, imaging scales (e.g., the Fazekas scale) can be used in quantitative evaluation—necessitating to strengthen the training of relevant scale knowledge for clinicians.

## 15 HEMORRHAGE

Acute neurological deficits caused by cerebral hemorrhage also need to be differentiated from acute ischemic cerebrovascular disease. The preferred imaging method is CT. However, clinicians may choose MRIs to simultaneously observe the presence of bleeding and new infarctions. In the super‐acute phase, a hemorrhage presents as a low signal on T1WI, or a high signal on T2WI, FLAIR, and DWI within 24 h. In the acute phase, all of the above sequences present as low signals within 1–3 days (Maizlin et al.; 2009, Dorenbeck et al.; 2005).

## 16 HYPOGLYCEMIA

Hypoglycemia episodes are common in ED visits. Patients may experience a series of neurological symptoms, including tremors, blindness attack, a consciousness disorder, and comas, which often mimic AIS and are, therefore, frequently misdiagnosed by clinicians. The imaging mainly characterizes cerebral edema with a high signal on T2WI, FLAIR, and DWI, or a low ADC value in the parietal lobe, occipital lobe, hippocampus, globus pallidus, and striatum (Bathla & Hegde, 2013, Arrabyru et al.; 2019, Aiba et al.; 2019)—a condition which is reversible and disappears with the recovery of blood glucose. Noteworthy, the thalamus and white matter are usually not involved. As blood glucose tests are routine, a diagnosis of hypoglycemia can be readily confirmed when using a combination of blood sugar level tests and the changes observed on MRIs.

## 17 SUMMARY

Patients with TNAs represent a challenge to emergencyclinicians. Although both CT and MRI have shown excellent performance in differential diagnosis, MRI can only be done in a delayed fashion especially in the emergency setting, and CT diagnosis is a critical measure because of its timely availability. Meanwhile, more detailed medical history and neurological examination are crucial for making a prompt and accurate diagnosis for treatment.

## AUTHOR CONTRIBUTIONS

Ying Wang––Conception and design; Administrative support, provision of study materials, collection and assembly of data; Data analysis and interpretation; Manuscript writing; Final approval of the manuscript. Hao Zha––Provision of study materials; Collection and assembly of data; Data analysis and interpretation; Manuscript writing; Final approval of the manuscript.

## CONFLICTS OF INTEREST

All authors have completed the ICMJE uniform disclosure form. The authors have no conflicts of interest to declare.

### PEER REVIEW

The peer review history for this article is available at https://publons.com/publon/10.1002/brb3.2780

## Data Availability

All data obtained or analyzed during this study are included within the article.
